# Visual learning with reduced adaptation is eccentricity-specific

**DOI:** 10.1038/s41598-017-18824-7

**Published:** 2018-01-12

**Authors:** Hila Harris, Dov Sagi

**Affiliations:** Department of Neurobiology, The Weizmann Institute of Science, Rehovot, 76100 Israel

## Abstract

Visual learning is known to be specific to the trained target location, showing little transfer to untrained locations. Recently, learning was shown to transfer across equal-eccentricity retinal-locations when sensory adaptation due to repetitive stimulation was minimized. It was suggested that learning transfers to previously untrained locations when the learned representation is location invariant, with sensory adaptation introducing location-dependent representations, thus preventing transfer. Spatial invariance may also fail when the trained and tested locations are at different distance from the center of gaze (different retinal eccentricities), due to differences in the corresponding low-level cortical representations (e.g. allocated cortical area decreases with eccentricity). Thus, if learning improves performance by better classifying target-dependent early visual representations, generalization is predicted to fail when locations of different retinal eccentricities are trained and tested in the absence sensory adaptation. Here, using the texture discrimination task, we show specificity of learning across different retinal eccentricities (4–8°) using reduced adaptation training. The existence of generalization across equal-eccentricity locations but not across different eccentricities demonstrates that learning accesses visual representations preceding location independent representations, with specificity of learning explained by inhomogeneous sensory representation.

## Introduction

Specificity is a well-known characteristic of visual learning^1,2^. For instance, enhanced visual sensitivity due to repeated training is limited to the trained retinal location^3,4^. Specificity, a robust psychophysical finding, was taken to reflect the site of learning within the visual processing stream, suggesting that plasticity within the primary visual cortex underlies visual learning^4,5^. However, additional results suggest also the involvement of multiple cortical areas^6–10^. An important difference between low and high level visual areas is spatial invariance; while in lower visual areas neuronal representations correspond to a limited visual field of view, with an eccentricity dependent scale, visual representations higher in the visual processing pipeline show spatial invariance^11^, better matching our subjective experience of space invariant recognition and “object constancy”^12^.

In recent years, an increasing body of evidence demonstrates conditions under which learning generalizes, for example, by applying a double-training paradigm or a brief pre-test under the transfer conditions^13–15^. Others found that learning generalizes during the initial phase of training^6^, when brief training is applied, when two locations are trained together^16^, or when the task is not too demanding^16^. In particular, it has been suggested that specificity results from sensory adaptation induced during training^17–19^. We have previously shown^18,20,21^, using the texture discrimination task, that performance decreases when the number of trials within a training session increases, and that the perceived orientation of a test line is changed^18^ (tilt after-effect), as expected from sensory adaptation^22^. Adaptation to oriented patterns^23^, a result of continuous exposure to an oriented pattern (e.g. gratings), was shown to be reduced when concurrently adapting to two orientations 45° apart^24^. Accordingly, we presented during training stimuli with line elements oriented 45° away from the target, showing reduced adaptation in the texture task and generalization to a new location^17,25^. The effectiveness of the reduced adaptation training was in accordance with several underlying adaptation features such as locality and orientation-selectivity^17^. Adaptation-driven specificity may account for previous research done in the field, since some of the conditions that resulted in generalization can be viewed as reduced adaptation conditions. Since adaptation is induced by repetitive stimuli, any modulation that reduces repetitions (brief training, presentation of different stimuli during training) is also likely to reduce adaptation^21,26,27^.

While previous studies suggest that adaptation interferes with learning^27,28^, of particular interest here is the suggestion made that adaptation interferes with *invariant* learning by adding network-dependent modifications in early visual areas^17^. According to this account of learning specificity, the specificity results from differences between the sensory representations (possibly in the primary visual cortex) of the trained and the transferred stimuli^29^. A readout mechanism^2,30^ that learns to perform the discrimination task at a fixed trained location, by capturing location-dependent specific features that happen to be useful for task performance at that location^31^, will fail to transfer this learning to a new location. Generalization is obtained when the stimulus evokes similar representations at the trained and the transfer locations, as is the case without adaptation when the two locations share the same eccentricity. However, for a constant target, the evoked low-level representations are expected to differ in the absence of adaptation when the trained and transferred locations differ in eccentricity, since the human visual system, including the retina and the visual cortex, is known to be retinotopic with a nonuniform spatial sampling^32–34^. Importantly, the sampling rate decreases with eccentricity, that is, fewer neurons are allocated to process the peripheral relative to the central visual field, and the size of their receptive field increases with eccentricity, producing a close to constant cortical population point image^35^. Thus, specificity of learning is predicted even in the absence of sensory adaptation if learning is based on low-level visual information, before location invariant object-features are abstracted. Finding transfer of learning across eccentricities may imply that the relevant performance limiting neuronal-representation is space invariant (that is, corrected for the low-level distortions), possibly corresponding to our actual (action relevant) perception of space^36,37^.

In the experiments reported here, the effects of adaptation were reduced by interleaving target trials with no-target trials oriented 45° away from the target (see above – ‘reduced adaptation’), previously shown to allow for transfer of learning across equal-eccentricity locations, using comparable experimental parameters^17,19,25,38^. Furthermore, training sessions were kept short (~30 minutes) in order to further minimize adaptation and fatigue^18,39^. Here we trained observers with the target positioned at one eccentricity (4°), after which they were trained with the target at another eccentricity (8°). The choice of eccentricities is motivated by results showing a relatively constant behavior between 4° and 8° of eccentricity^40,41^. The results point to eccentricity-dependent specificity of learning.

## Methods

### Apparatus

**Figure 1:**
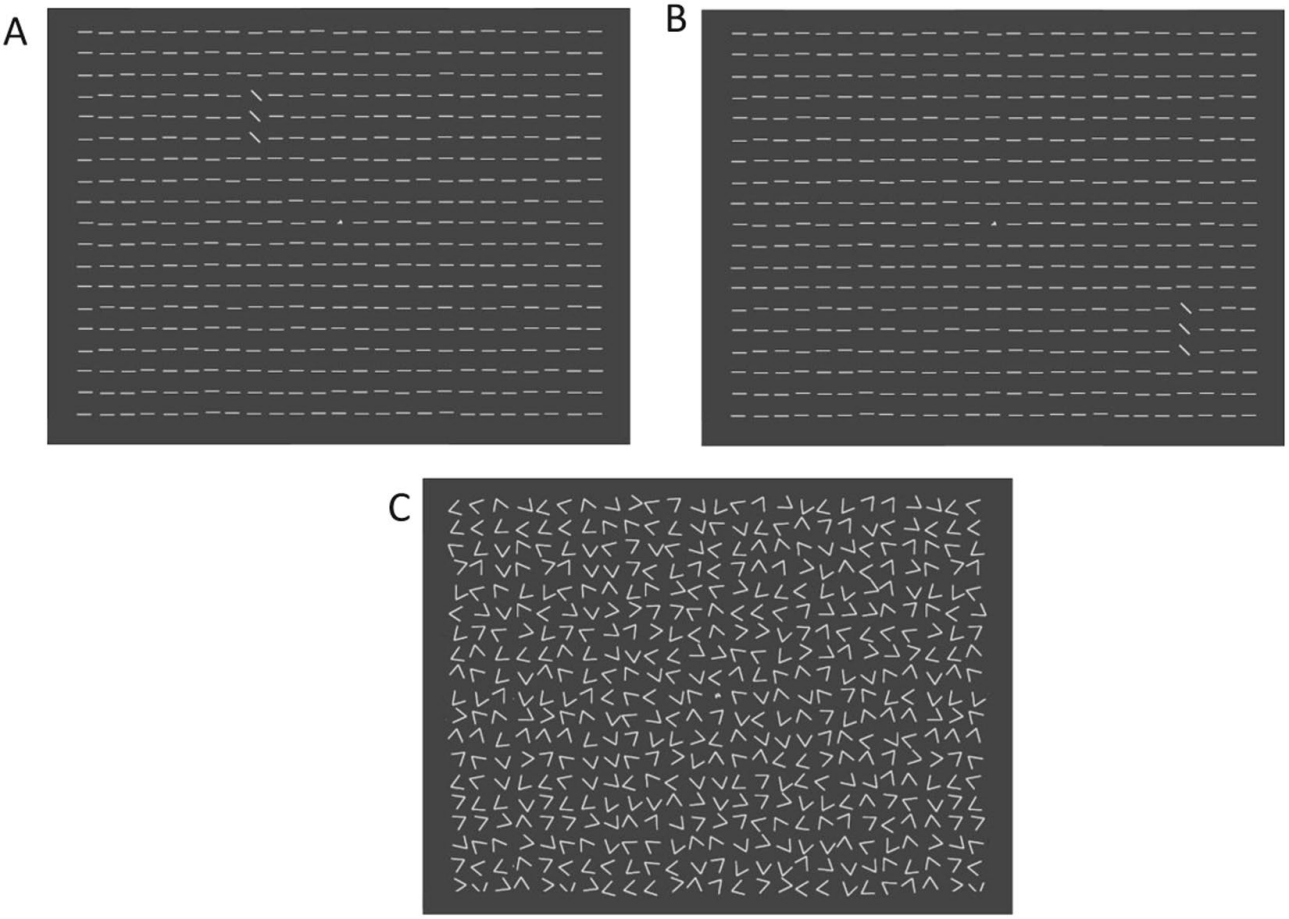
Texture Discrimination Task. **(A)** Target stimulus with diagonal lines at 4° eccentricity. **(B)** Target stimulus with diagonal lines at 8° eccentricity. Subjects had to determine (1) the letter at the center of display (T or L), and (2) the arrangement of the three diagonal lines (horizontal or vertical). **(C)** Mask stimulus.

Stimuli were presented on a 19″ Mitsubishi Diamond Pro 930SB color monitor, using a PC with an Intel processor. The monitor refresh rate was 100 Hz. The luminance of the stimulus (line textures) was 63–65 cd/m^2^, drawn on a (close to) black background (see Fig. 1), thus with Michelson contrast = ~1. The experiments were carried out in a dark environment.

### Stimuli and task

Observers were trained with the standard texture discrimination task (TDT^4^) using a modified stimulus (Fig. 1). In the original experiment^4^, a typical texture experiments, there was a 3 × 1 target array embedded in a 19 × 19 stimulus array, that is 361 locations out of which 3 were occupied by target elements, 357 by background elements, and one location at the display center was occupied by a fixation letter (‘T’ or ‘L’, randomly oriented). Here, background elements were added so that instead of a 19 × 19 array^17^, a 19 × 25 (H × W) array was presented. Texture elements were short lines, 0.5° × 0.035°, spaced 0.72° apart, with 0.05° jitter. Targets lines were diagonal (45°) while background lines were horizontal. The display size was 14° by 18.5° of visual angle, viewed from a distance of 100 cm. Observers had to judge the arrangement of the target triplet (an array of three diagonal lines) and report whether it was horizontal or vertical. The peripheral target was embedded in the background and its position was centered at 4° or 8° of a visual angle (depending on the experiment) relative to the center of display (Fig. 1). Mask patterns were 19 × 25 arrays of randomly oriented ‘V’-shaped patterns, with the central one (at fixation, replaced by a randomly oriented superposition of the two possible fixation targets (‘T’ and ‘L’). The target frame was presented for a duration of 10 ms and the mask frame for 100 ms duration.

Each trial was self-initiated by the observer. Fixation was enforced at the center of the display by a forced-choice letter discrimination task between a randomly oriented ‘T’ and a randomly oriented ‘L’ presented with the target (10 ms). Next, the observer had to report whether the peripheral target array was horizontal or vertical. Responses were provided by pressing a computer mouse click. Auditory feedback was provided if there was an incorrect response for the fixation (T\L) task. Target and mask stimuli were separated by a time interval (stimulus onset asynchrony, SOA) ranging from 20 to 400  ms (15 values: 20–220 in steps of 20 ms, 260, 300, 340, 400 ms). In each daily session, SOA was randomized across trials, with 18 trials per SOA^17^. The measured psychometric functions (%correct discrimination as a function of SOA) were fitted with the Weibull function (using a maximum likelihood estimation) in order to estimate the discrimination threshold^20^

1 $$P(t)=\frac{1}{2}[1+(1-fe)(1-{e}^{-{(\frac{t}{T})}^{\beta }})],$$

where *P(t)* is the measured probability of a correct response, t represents the varied experimental parameter (SOA in msec), T is the estimated discrimination threshold for each session, β describes the psychometric function’s estimated slope, and *fe* is the estimated “finger error” parameter (0 ≤ *fe* ≤ 1). Performance on the T/L task was analyzed separately. The experimental results were analyzed in two ways: (1) whole session estimation, where fitting was done to each observer data using all trials in a session (N = 270 trials), and (2) sessions were divided into 3 ordered sub-sessions with each sub-session fitted separately (N = 90 trials).

### Observers

Fifteen observers participated in the experiments reported here, with 6 additional observers participating in an experiment reported in the Supplementary Information. All had normal or corrected-to-normal vision. Observers were naïve to the purpose of the experiments, and gave their written informed consent. The work was carried out in accordance with the Code of Ethics of the World Medical Association (Declaration of Helsinki), and approved by the local Institutional Review Board of the Weizmann Institute.

### Procedures

All observers received pre-training trials with SOA = 360 ms prior to the first training session on the first day. During this pre-training phase, the observers achieved a criterion of 10 (but see below, 5–8° training group) correct trials in a row (for both fixation and peripheral target tasks, which required a total of ~30 trials). Observers were informed about the change in the target’s position prior to the location change, but there was no pre-training at the new location. In all experiments reported here, adaptation was reduced by interleaving no-target trials, presenting stimuli consisting of uniform textures with lines tilted 45° from the target line^25^. These trials were equal in number to the test trials (N = 270), effectively doubling experimentation time, and were randomly intermixed during each experimental session. Observers were required to provide a target-response on all trials, including the no-target trials (termed ‘dummy trials’), resulting in chance performance.

#### 4–8° training group

Observers (n = 5) trained the texture discrimination task (see above) for four daily sessions with the target positioned at 4° (upper-left quadrant, azimuth of −45°), after which they were trained at 8° (lower-right quadrant, azimuth of 119°) eccentricity. Two observers of this group did not complete the experiment and performed only one session at 8° eccentricity, whereas three observers were trained at this eccentricity for 4 sessions.

#### 8° novice group

Observers (n = 6) having no previous experience with the texture discrimination task, trained the task for one daily session with the target positioned at 8° eccentricity (lower-right quadrant, azimuth 119°).

#### 5–8° training group

Observers (n = 4) that were trained and tested earlier^25^ with targets presented at 5° eccentricity (azimuth −45 followed by 135°) using the standard texture discrimination stimulus of a 19 × 19 array, were trained here using the extended stimulus (19 × 25 array) with the target at 8° eccentricity (lower-right quadrant, azimuth 119°). All these observers were previously trained with a reduced adaptation method (with interleaving dummy trials, as described above), two were trained with the gradual SOA method (monotonically reducing SOA during the training session) combined with random SOA tests, and two were trained with the random SOA method but with reduced pre-training (a criterion of 5 trials instead of 10 as described above, as detailed in Harris and Sagi^25^).

## Results

To verify that the reduced-adaptation method used here at 4° eccentricity was indeed effective, learning curves were compared with two types of curves obtained in our previous study^17^, fortified by some unpublished data (Supplementary Fig. 1), using (1) the reduced adaptation training method (N = 13; Harris *et al*.^17^, +45 method), and (2) the standard training method (N = 7; Harris *et al*.^17^, standard method). This comparison, shown in the Supplementary Information, confirms the effectiveness of the reduced-adaptation method used here.

**Figure 2:**
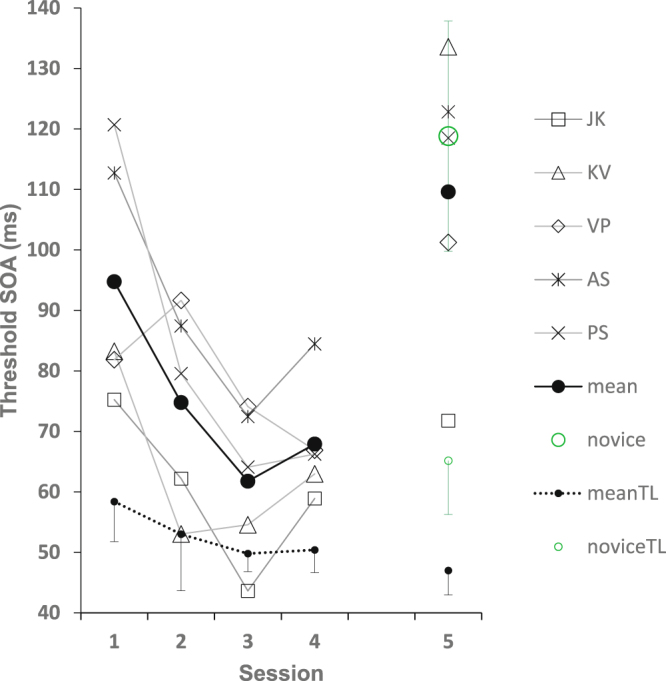
Five observers trained with the texture discrimination task over four daily sessions with the target positioned at 4° (sessions 1–4), after which they were tested at 8° eccentricity (4–8° eccentricity group). All observers show learning at the 4° eccentricity (sessions 1–4), followed by increased thresholds at 8° eccentricity (session 5). Their thresholds on session 5 are comparable to that of the novice group having no previous experience (8° novice group) denoted by O(±SE); thus learning is eccentricity specific. Thresholds were computed by fitting a Weibull function to whole session results (270 trials distributed over 15 SOA values). Results are also presented for the fixation T/L task (mean ± SE), showing experience dependence, but no effect of target eccentricity.

The 4–8° eccentricity group exhibited significant learning following training (Fig. 2; data also shown in Supplementary Fig. 1 as *Adapt- 4*, showing main effect of training day). At the 4° eccentricity, all observers showed learning, thresholds improved from 95 ± 9 (session 1, mean ± SE) to 65 ± 5 ms (sessions 3–4, mean ± SE) during the four days of training (pairwise t-test, session 4 vs 1, t(4) = 3.69, p = 0.01). On the 5th day, with the target at 8° eccentricity, all observers had their threshold at the new location increased, with a group mean of 110 ± 11 ms comparable to that of day 1 (mean ± SE, pairwise t-test: session 5 vs 4: t(4) = 4.34, p < 0.01; session 5 vs session 1: 2-tailed pairwise t-test, t(4) = 1.51, p = 0.2), which indicates specificity of learning. This specificity is further supported by the results of the 8° novice group. This group, with no previous experience with the task, was trained during one session with the target at 8°, that is, the transfer condition of the 4–8° eccentricity group. The average novice-group threshold was 118 ± 20 ms (mean ± SE, compared with 110 ± 11 ms of the trained 4–8° eccentricity group; 2-tailed t-test: t(9) = 0.37, p = 0.72), which confirms that the learning of the eccentricity group was specific to the trained location, that is, training at 4° has no effect on performance at 8°. Therefore, unlike previous reports showing generalization across equi-eccentric locations of learning with reduced adaptation training, here, when the target eccentricity is changed, the learning is specific.

**Figure 3:**
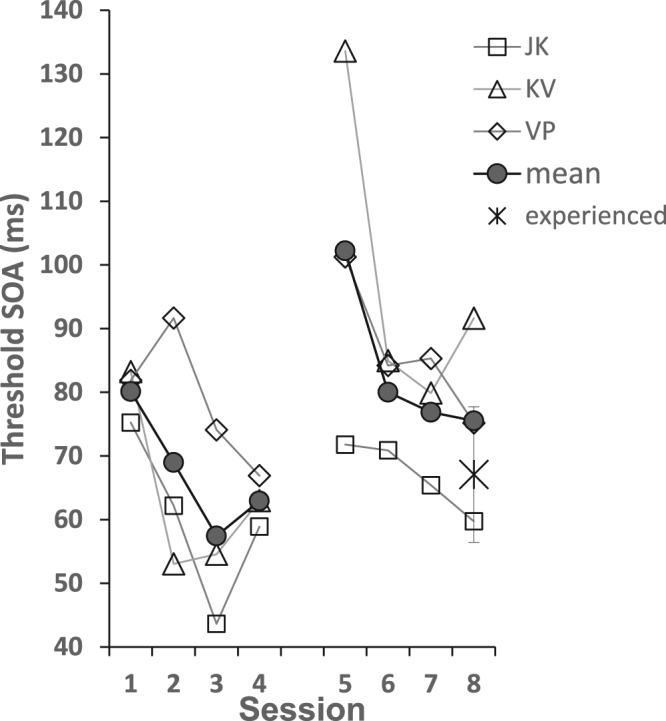
Three observers from the 8° eccentricity group (JK, KV, VP) were trained at 8° eccentricity (sessions 5–8, round symbols), after four sessions of training with the target positioned at 4° (sessions 1–4), showing learning at 8° eccentricity. The final performance at 8° is not significantly different from that of the experienced group (5–8° training group, N = 4) denoted by the X(±SE). Thresholds were computed by fitting a Weibull function to whole session results (270 trials distributed over 15 SOA values).

**Figure 4:**
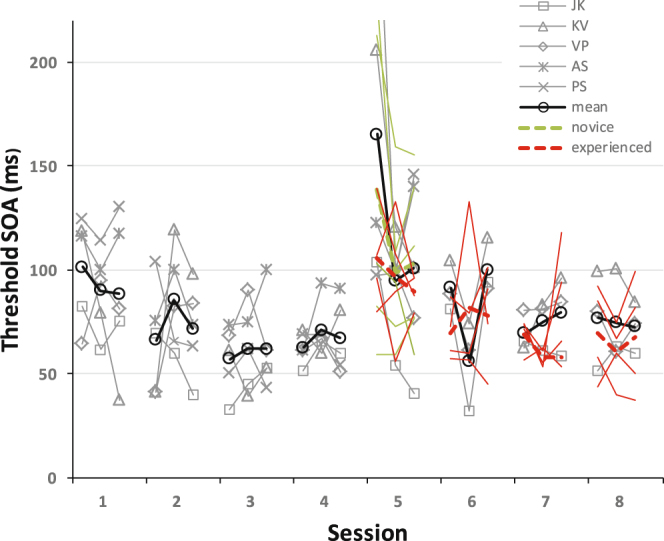
Detailed results showing thresholds of individual observers from the 4–8 training group, and their means (black curves), at 3 time points during each session. The different observers are marked by different symbols. Also shown are the mean and individual results of the 8-novice group (green curves; dashed for group mean, continuous for individual obs.) and of the 5–8 training group (red curves; dashed for group mean, continuous for individual obs.). The 4–8 training group show specificity (N = 5), demonstrated by the increased initial thresholds on day 5 relative to day 4 in all observers. Their thresholds were similar to that of the novice group on the transfer day (day 5), and to that of the experienced group at the end of testing (day 8). Thresholds were computed by fitting a Weibull function to 1/3 session results (90 trials each, 15 SOA values).

Additional support for the specificity of learning is provided by learning at the new location. Three observers of the 4–8° eccentricity group continued training with the target at 8° eccentricity (Fig. 3). Thresholds decreased at the new location (8°) during sessions 5–8, showing re-learning (102 ± 17 to 76 ± 9 ms, mean ± SE,pairwise t-test: t(2) = 3.09, p = 0.045). We have also trained at 8° eccentricity observers previously trained at 5° eccentricity^25^. The 5–8° training group had an initial threshold of 102 ± 10 ms at 8° (mean ± SE), comparable to the initial thresholds of the other two groups at that eccentricity (8°). This threshold improved during the four training sessions at 8° to a level of 67 ± 10 ms (mean ± SE, pairwise t-test: t(3) = 2.75, p < 0.035; Fig. 4), comparable to that of the 4–8° eccentricity group (76 ± 5 ms; 2-tailed t-test between means of sessions 7–8: t(5) = 0.43, p = 0.68). Although the initial threshold of this group, though in complete agreement with the present results (see also Fig. 4), does not clearly indicate the specificity vs generalization issue, due to the diverse training methods experienced by this group (multiple locations, varied pre-training), we believe that their final threshold, obtained using the same method used with the present experimental groups, expresses the full training potential at this eccentricity.

Figure 4 presents more detailed data. Shown are the individual observers’ thresholds at three time points along each session (computed from 90 trials each), and their corresponding means. Learning at 4° is evident by the improvement in threshold between the first testing and the last at that eccentricity (mean of 34 ms, range of 14 to 71 ms, pairwise t-test: t(4) = 3.43, p = 0.013). Specificity is indicated by the increased threshold at 8°, on day 5, where, for all observers, the first measurement yielded an increased threshold relative to the last measurement at 4° (mean of 98 ms, range of 44 to 247 ms, pairwise t-test: t(4) = 2.41, p = 0.037). The fast within session learning on day 5 is in agreement with previous experiments showing specificity in texture discrimination learning^17^, and with the phenomenology of perceptual learning^42^. Specificity is supported by the quasi-equality between thresholds of the 4–8° eccentricity group (N = 5) and a novice group (N = 6) trained only at 8° (shown here on day 5, the visible difference between the initial thresholds on that day is not statistically significant; 2-tailed t-test: t(9) = 0.57, p = 0.58). This result indicates perfect specificity, where learning at the first location (4°) does not facilitate, or speeds up^17,43^, training at the new location (8°). At the end (day 8), the last measurements show no statistical difference (p = 0.78, 2-tailed t-test, t(5) = 0.3) between the experienced group (N = 4) and the 4–8° eccentricity observers (N = 3) that made it to the end (there is also no significant difference between the other two measurement pairs corresponding to these two groups on day 8, p = 0.7, t(5) = 0.41, and p = 0.36, t(5) = 1.01, 2-tailed t-test).

## Discussion

Observers trained under reduced adaptation conditions failed to generalize their learning to a new eccentricity. At the new eccentricity, their mean performance equaled that of a previously untrained (novice) group. Specificity is supported by renewed learning at the new eccentricity, converging to a level corresponding to that of highly trained observers (the experienced group). Previous results obtained with this method (training trials interleaved with uniform textures tilted 45 from the target, relatively short sessions) showed complete transfer of learning to a new location when having eccentricity equal to that of the trained location^17,25^.

The results agree with predictions made by a theory assuming learning involves acess to low-level visual representations, before location invariant object-features are abstracted (see Introduction). Specificity of learning is predicted in the absence of sensory adaptation if the trained neuronal representation differ from the tested representation at transfer. This limitation of learning is expected from any learning mechanism which learns from examples, more so when the number of examples is small as in a typical perceptual learning experiment. Perhaps the most general rule can be derived from statistical learning, in the form of overfitting^1^. The task of modeling visual inputs, as required in learning experiments, is largely similar to a statistical modeling problem. Overfitting happens when a limited discrimination set is employed during training, allowing to model the data using parameters highly useful for the specific training set but of limited generality (not useful with untrained samples). Here, one wants to fit a model to the visual data using stimulus and task relevant parameters, however other incidental parameters^29^ introduced by the training paradigm, and by neuronal encoding, may facilitate discrimination and enter the model. This would lead to a model that represents training dependent features rather than stimulus- and task-relevant features. For example, training with a stimulus at a fixed location^4^, or of a fixed contrast^44,45^, may encourage the learning mechanism to base discrimination on properties of neuronal representations specific to that particular location or contrast trained^45^. On the other hand, variations in stimulus parameters during training, such as with multiple targets, may force the learning mechanism to base discrimination on more general representations^46^. Importantly, overfitting does not impose any neural implementations in terms of the site of learning, but rather refers to the encoded information available to learning. Within this theoretical framework, our current results imply that learning can access low-level eccentricity dependent information.

Retinal and cortical (CMF) inhomogeneity imposes dissimilar representations (i.e. cortical response patterns) for different retinal eccentricities, and correspondingly generates eccentricity-dependent encoding. With repeated training at one eccentricity, the learned model (template, classifier) becomes strongly fitted to the spatial characteristics of that location, leading to overfitting. Therefore, a classifier trained at one eccentricity is useless when dealing with information encoded using a different receptive-field structure, as with a different eccentricity. Our results suggest that generalization is constrained by the receptive field size and cortical magnification factor (CMF) at an early stage of visual processing where the visual field topography is preserved. Another possibility is that lateral interactions underlying texture discrimination^47^ vary with eccentricity^48,49^. Importantly, these results indicate that adaptation does not necessarily play a unique role in determining specificity. Although we believe that adaptation is a leading source of variability in the neuronal representations stimulated during training, and consequently, of specificity of learning, here we provide evidence for other sources of variability.

Our results suggest the possibility of independent learning of stimulus/task-related information and of CMF. Of particular interest here are reports showing transfer of Vernier learning across locations of different eccentricity using the double-training method^15^. Wang *et al*.^15^ found such a transfer for a Vernier task coupled with a motion-direction discrimination task, but not when coupled with a contrast discrimination task. Thus, when training with two tasks, generalization is constrained by the less specific task (e.g., motion-direction discrimination), implying that the extended CMF properties available to the less specific task can be applied to the more specific task. Similarly, transfer of orientation discrimination learning across different eccentricities after pre-testing at the eccentricity to be transferred to^14^, can also be explained by a learning process that captures the CMF corresponding to the new location during the pre-test.

Here we address learning and cortical organization by testing transfer across different eccentricities in the visual field. Previous work suggested that the specificity of visual learning results from sensory adaptation. Generalization was obtained across symmetrical retinal locations for a given eccentricity. According to the proposed adaptation-dependent learning model, learning involves a low-level adaptable visual network representing stimuli features, and a higher level classifier performing the discrimination task. Unadapted networks can generalize learning, whereas adaptation adds variability to the lower-level visual networks. A readout mechanism that learned (and was overfitted to) one adapted location will fail to generalize. According to this view, local adaptation is suggested to increase spatial variability within the encoding stage, leading to distinct neuronal populations encoding stimuli differently. However, there may be other sources of variability in visual training. Whereas adaptation is a functional source of variability (a consequence of the repetitive training), eccentricity is an anatomical source of variability. The CMF accounts for a considerable amount of performance variation in several visual tasks^50,51^, but the induced scale variations are thought to be dynamically corrected, though not perfectly, for object size^52–54^ (size constancy). Our results here suggest that some eccentricity dependent neuronal parameters, masked by perceptual constancies, are available to visual learning.

## Electronic supplementary material

Supplementary Information
