# Supplementary material for: Visual learning with reduced adaptation is eccentricity-specific
Source: Sci Rep. 2018 Jan 12;8:608. doi: 10.1038/s41598-017-18824-7 (PMC5766564; doi:10.1038/s41598-017-18824-7)
Supplement: Supplementary file 1 — Supplementary Information [file 41598_2017_18824_MOESM1_ESM.pdf]

Visual learning with reduced adaptation is eccentricity-specific

Hila Harris and Dov Sagi

Department of Neurobiology,

The Weizmann Institute of Science, Rehovot, 76100 Israel

Supplementary Information

Individual learning curves describing results from the first 4 training sessions (location 1), obtained using the 3 methods: reduced adaptation at 4° (N=5), reduced adaptation at 5° (N=13), and the standard method at 5° (N=7) are depicted in Supplementary Fig. 1. The latter two experimental groups include observers from previous experiments described in Harris et al (2012)[1], as well as from yet unpublished experiments (6 out of 13 in the reduced adaptation method at 5°) following the same Methods. Individual learning curves differ in their starting point, in agreement with previous studies[2-4], however, the results clearly show that the thresholds measured here with 4° target location overlap with those previously obtained[1] at 5°, using the same paradigm but with a smaller texture array (19×19 vs 19×25, see Methods). On the other hand, there is a very small overlap between the current thresholds and those previously obtained[1] with the standard method. Statistical analysis shows a highly significant main effect of experimental condition and day (mixed design ANOVA with one within and one between factors; effect of training method: $F(2,22)=6.7$, $p=0.005$; effect of day, $F(3,66)=33.1$, $p<0.0001$; method × day interaction, $F(6,66)=7.36$, $p<0.0001$). A post-hoc analysis showed that the current thresholds do not differ significantly from our previous thresholds obtained with the reduced adaptation method ($F(1,16)=0.48$, $p=0.5$; effect of day, $F(3,48)$, $p<0.0001$; method × day interaction:

$F(3,48)=1.47$, $p=0.23$), but are significantly different from the thresholds obtained with the standard condition ($F(1,10)=7.96$, $p=0.018$; effect of day $F(3,30)=20.38$, $p<0.0001$; method × day interaction: $F(3,30)=6.14$, $p=0.002$). Thus, the effectiveness of the reduced-adaptation method is confirmed.

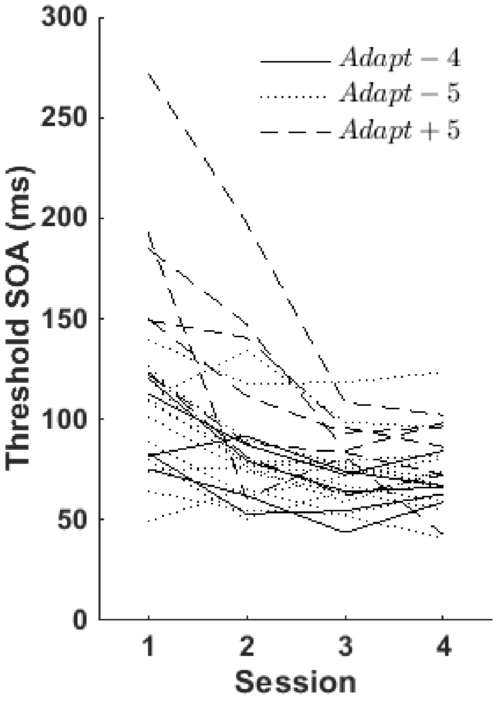

Supplementary Fig. 1: *A comparison between the current training results (4°, sessions 1-4, location 1, marked by Adapt- 4, continuous curves) and results from previous studies[1] (5°, reduced texture width) using the standard (Adapt+ 5, dashed curves) and the reduced adaptation methods (Adapt- 5, dotted curves). Each curve represents data from a single observer. The 5° results were obtained using the same stimuli and procedure used here, except for target location and texture background width (full methods described in Harris et al[1], +45 and standard methods).*

1. Harris, H., Gliksberg, M. & Sagi, D. Generalized perceptual learning in the absence of sensory adaptation. *Curr Biol* **22**, 1813-1817 (2012).
2. Harris, H.*, et al.* Response: Commentary: Perceptual learning in autism: over-specificity and possible remedies. *Frontiers in integrative neuroscience* **10**, 36 (2016).
3. Fahle, M. & Henke-Fahle, S. Interobserver variance in perceptual performance and learning. *Invest Ophthalmol Vis Sci* **37**, 869-877 (1996).
4. Yehezkel, O., Sterkin, A., Lev, M., Levi, D.M. & Polat, U. Gains following perceptual learning are closely linked to the initial visual acuity. *Scientific reports* **6**, 25188 (2016).
